# Isolation and Identification of *Aspergillus* spp. from Rotted Walnuts and Inhibition Mechanism of *Aspergillus flavus* via Cinnamon Essential Oil

**DOI:** 10.3390/foods14030357

**Published:** 2025-01-22

**Authors:** Doudou Zhang, Kangjing Luo, Shaocong Wen, Qing Zhou, Bochao Li, Wenhui Liang, Jianbing Di

**Affiliations:** 1 Food Science and Engineering College, Shanxi Agriculture University, 1 Mingxian South 3 Road, Taigu District, Jinzhong 030801, China; 15698543093@163.com (D.Z.); 18295992707@163.com (K.L.); 13934714216@163.com (B.L.); 15502272127@163.com (W.L.); 2 Shanxi Fruit and Vegetable Storage and Processing Technology Innovation Center, 1 Mingxian South 3 Road, Taigu District, Jinzhong 030801, China

**Keywords:** walnut, *Aspergillus* spp., isolation, identification, cinnamon essential oil

## Abstract

Walnuts are prone to contamination by rotting fungi. However, the microflora present in walnuts across various regions of China has not been thoroughly investigated. Cinnamon essential oil (CEO) is commonly used in food preservation because of its natural safety and strong antimicrobial properties. Additionally, studies on the antifungal potential of CEO to prevent walnut spoilage are limited. Therefore, we investigated *Aspergillus* spp. contamination in moldy walnuts stored across different locations in Shanxi, China. A total of 100 moldy walnut samples underwent traditional mycological analysis to isolate *Aspergillus* spp. The antibacterial properties and the mechanisms by which CEO targets *Aspergillus* spp. were thoroughly investigated. Five representative morphospecies were subsequently classified to the species level using Internal Transcribed Spacer sequence analysis. The dominant species were *Aspergillus flavus* and *Aspergillus fumigatus*, with frequencies of 100% and 93%, respectively, followed by *Aspergillus nigers*, *Aspergillus terreus*, and *Aspergillus tubingensis*, with frequencies of 78%, 47%, and 40%, respectively. Overall, 358 fungal species belonging to the *Aspergillus* genus were recovered. The MIC of CEO against *A. flavus* in vitro was 0.78 g/L. Furthermore, CEO compromised the permeability and integrity of the cell membrane, causing the leakage of intracellular components and promoting the accumulation of malondialdehyde compounds and a decrease in superoxide dismutase activity. Overall, we isolated and identified *Aspergillus* spp. in moldy walnuts and confirmed the feasibility of using CEO as a green anti-*Aspergillus* spp. agent for the preservation of walnuts.

## 1. Introduction

Walnut (*Juglans regia* L.) holds both economic and medicinal value and is an important dry fruit and woody oilseed [1]. The major walnut-producing countries include China, the United States, Chile, Iran, Ukraine, Turkey, France, and India [2]. According to the Food and Agriculture Organization of the United Nations (2016), China is the leading producer of walnuts, cultivating 400,000 hectares in 2016 and yielding 1.79 million tons of in-shell nuts, which represented 47.7% of the global walnut production. Walnuts are rich in unsaturated fatty acids, proteins, vitamins, phytosterols, and tocopherols [3]. These nutrients modulate immune function and inflammatory responses, help to prevent cancer, and reduce the risk of diabetes and liver disease in humans [4,5,6,7]. Walnuts are prone to contamination by rotting fungi, including *Alternaria* sp., *Aspergillus* sp., *Fusarium* sp., *Mucor* sp., *Monilinia* sp., *Penicillium* sp., and *Rhizopus* sp. [8,9,10]. In a study conducted in Iran, a researcher isolated 144 strains of *Aspergillus flavus* from dried nuts and grains [11]. *Aspergillus* spp. are among the three genera of fungi responsible for nut spoilage and toxin production [12,13]. The toxins produced by *Aspergillus* spp. pose significant health risks across all stages of human life. They are carcinogenic and immunosuppressive, impair placental and fetal growth and development, can result in disrupted protein synthesis, and affect the metabolism of micronutrients [14]. Thus, identifying various *Aspergillus* species present in walnuts from the Shanxi regions is crucial for developing guidelines to improve walnut storage and preservation.

Traditional methods for inhibiting the growth of *Aspergillus* spp. in walnut products include physical, chemical, and biological treatments [15,16,17]. In recent years, a range of biologically derived fungicides, including botanical extracts (comprising active phytochemicals and essential oils), plant growth promoters, mineral salts, and antagonistic microorganisms, have been successfully applied to mitigate postharvest spoilage in fresh produce [18,19]. Among them, important oils from spices, such as cinnamon essential oil (CEO), are frequently used for food preservation, owing to their natural safety and strong antimicrobial properties [20,21,22]. Numerous studies have revealed the antimicrobial properties of CEO against different pathogenic bacteria and spoilage microorganisms [23]. Ref. [24] showed that CEO efficiently suppressed both mycelial growth and *aflatoxin* production. The minimum inhibitory concentration (MIC) of CEO against *A. flavus* is 3.125 μL/mL [25].

Although walnuts are extensively produced and consumed in China, with a notable risk of mycotoxin and fungal contamination, limited research has explored the antifungal properties of CEO for preventing walnut spoilage. Additionally, the mycoflora present in walnuts across various regions of China have not been thoroughly investigated. Therefore, the aims of this study are as follows: (i) to investigate moldy walnut samples randomly collected in Shanxi, China, and identify the species of *Aspergillus* present; (ii) to evaluate the antimicrobial effects of CEO against the *Aspergillus* spp. dominant strain in walnut products; and (iii) to elucidate the mechanism by which CEO exerts its antifungal effects, particularly focusing on the disruption of cell membrane integrity and oxidative stress evaluation.

## 2. Materials and Methods

### 2.1. Chemicals and Reagents

Potato Dextrose Agar (PDA) and Rose Bengal Agar (RBA) media were purchased from Beijing Solarbio Science & Technology Co., Ltd. (Beijing, China); a fungal DNA extraction kit was obtained from Sangon Biotech (Shanghai) Co., Ltd. (Shanghai, China); CEO was sourced from Guangzhou Jingjing Biological Technology Co., Ltd. (Guangzhou, China); and a superoxide dismutase (SOD) assay kit was acquired from Nanjing Jianjian Bioengineering Institute (Nanjing, China).

### 2.2. Locations and Sampling

Moldy walnut samples for fungal analysis were collected from the primary walnut-producing regions in Shanxi, China. Specifically, 100 moldy walnut samples with varied morphological traits were collected from storage facilities in Taigu, Shanxi, China (37°25′17″ N 112°33′03″ E). The contaminated samples were placed in pre-sterilized plastic bags and stored in a refrigerator at −80 °C to prevent any alterations until further analysis could be conducted. The monthly precipitation and average temperature data at the time of walnut collection are provided in Table 1.

### 2.3. Morphological Identification

Species identification was carried out using the methods outlined earlier [26,27]. The moldy walnuts were placed on an ultra-clean bench, and 25 g of the moldy walnut samples were placed into a conical flask that held 225 mL of sterile water. The combination was agitated thoroughly and oscillated with an oscillator for 1 h to create a suspension with dilution gradients of 10^−1^, 10^−2^, 10^−3^, 10^−4^, 10^−5^, and 10^−6^. A 1 mL sample of each dilution was then poured onto an RBA plate. This procedure was repeated thrice for each sample to obtain an average value. Simultaneously, 1 mL of sterile water was added to three separate sterile plates as a blank control. The dishes were kept in an incubator at 28 ± 1 °C, and the results were observed and recorded until day 5 of incubation. The color and morphology of the mold colonies were observed. The diameter of the colonies was measured, and the growth of each mold was recorded at 12 h intervals. Slides were prepared by picking the edge mycelium of each colony, and the morphology and spore structure of the mold spores were observed using a light microscope. Morphological identification was performed based on colony morphology, conidial structure, and growth characteristics on PDA. Specific features such as conidial size, shape, and color were recorded and compared with known characteristics of *Aspergillus* spp. to aid in species identification.

### 2.4. DNA Isolation, PCR Amplification, and Sequence Analysis

Total DNA from the fungi was extracted by picking a small amount of mycelium from a single colony grown on the plate, following the standard protocol of the Fungal Genome DNA Extraction Kit. Molecular identification of the fungi was performed using the primer pairs ITS1 (5′-TCCGTAGGTGAACCTGCGG-3′) and ITS4 (5′-TCCTCCGCTTATTGATATGC-3′). The PCR amplification conditions were as follows: pre-denaturation at 95 °C for 30 s, denaturation at 95 °C for 10 s, annealing at 60 °C for 5 s, and extension at 68 °C for 5 s. A total of 34 cycles were conducted, with the final extension step at 68 °C for 1 min. PCR products were verified by 1% agarose gel electrophoresis, and positive samples were sent to Parsons Brinckerhoff Biotechnology for sequencing. The obtained sequences were then submitted to the NCBI database (https://www.ncbi.nlm.nih.gov/, accessed on 15 November 2024) for Basic Local Alignment Search Tool (BLAST) analysis. Sequences with 100% identity were considered for species identification. In cases where multiple species showed 100% identity, additional morphological characteristics were used to differentiate between species. A phylogenetic tree was constructed using the Maximum Likelihood method with 1000 bootstrap replicates to assess the robustness of the tree topology. The tree was rooted with a closely related outgroup species to provide a reference point for the phylogenetic relationships.

### 2.5. Pathogenicity Testing

Pathogenicity assays were conducted to determine if walnut spoilage was caused by pre-isolated and purified strains of *Aspergillus* spp. Healthy dried walnut fruits were soaked in 75% alcohol for 30 min and rinsed five times using sterile distilled water. The fruits were then exposed to ultraviolet irradiation for 1 h on an ultraclean workbench and allowed to air dry. Spore suspensions (10 µL) were inoculated into sterilized walnut fruits and placed into Petri dishes. The non-inoculated dried walnut fruits were assigned to the control group and maintained in a steady temperature and humidity incubator at 28 °C for 5–10 days to observe mildew development.

### 2.6. Measuring the Antifungal Effectiveness of CEO

The strains used in the following experiments were derived from the isolation of *A. flavus* (strain C14) from decayed walnuts.

#### 2.6.1. Assay for the Inhibition of Mycelial Radical Growth

The antifungal activity of CEO was assessed using a previously established method [28] with minor adjustments. The CEO was first dissolved in DMSO, then thoroughly incorporated into PDA at 45 °C, and added to Petri dishes to achieve the desired final concentrations of 1, 0.75, 0.5, 0.25, 0.13, and 0 g/L. Distilled water and DMSO mixed with PDA served as control substances. Discs measuring 0.8 cm in diameter and excised from the edges of 10-day-old fungal colonies cultivated on PDA, were placed in the centers of sterile Petri dishes and incubated at 28 °C. Colony sizes were measured every 24 h along two intersecting axes, and the mean of these measurements was calculated for analysis.

#### 2.6.2. MIC and Minimum Fungicidal Concentration (MFC)

The MIC of the CEO for each strain tested was assessed using the broth microdilution technique, with minor adjustments, as outlined by [29]. The CEO was dissolved in a 5% Tween 80 solution. A volume of 100 μL of CEO dilutions, with concentrations ranging from 0.2 to 100 g/L, was added to 96-well plates, along with spore suspension. After a 48 h incubation at 28 °C, 15 μL of 0.1% resazurin solution was added to each well, and the plates were incubated in the dark for another 24 h. A color change from blue (oxidized state) to pink or colorless (reduced state) indicated fungal presence. The lowest concentration of CEO that entirely inhibited mycelial growth was recorded as the MIC.

Afterward, the mixture from wells that exhibited no fungal growth was transferred onto PDA plates. The MFC was identified as the smallest amount of CEO that fully prevented fungal growth within a 48 h period.

### 2.7. Sterilization Mechanisms

#### 2.7.1. Treatment of *A. flavus* Mycelium with CEO

In a sterile environment, 1 mL of *A. flavus* spore suspension was mixed with 90 mL of PDB liquid medium and incubated in a temperature-controlled shaker at 28 °C, rotating at 180 rpm for a duration of 3 days. Subsequently, 10 mL of CEO solution at varying concentrations was added, while an equal volume of distilled water was used for the control group. Following another 3 days of incubation, samples were obtained and filtered, and the mycelium was harvested. The collected mycelium was washed three times with sterile water and then kept at −80 °C for later examination. For analysis, 0.5 g of mycelium was mixed with 3 mL of 0.05 mol/L Tris-HCl buffer (pH 7.5), homogenized on ice at 4 °C, and centrifuged at 12,000 rpm for 20 min. The supernatant was gathered for additional analysis as the test sample.

#### 2.7.2. Cell Membrane Permeability

The permeability of the cell membrane was evaluated using the technique outlined by [30]. Fungal mycelium was cultured in PDB medium with shaking for 3 days, then separated onto agar and dried to remove moisture. Two types of pathogenic fungi, each with 1.5 g of mycelium, were divided into six portions and added to deionized water containing CEO at concentrations of 1.56, 0.78, 0.39, 0.20, and 0 g/L. The control group was given the same volume of deionized water. Conductivity measurements were recorded at 0, 1, 2, 4, 6, and 8 h at 25 °C. The changes in conductivity were monitored to evaluate the effects of CEO on cell membrane permeability.

#### 2.7.3. Cell Membrane Integrity

Changes in the integrity of cell membranes following treatment with CEO were investigated utilizing the approach mentioned earlier [31]. As outlined in Section 2.7.1 (the method of preparation of the supernatant), the absorbance of the supernatant was recorded at 595 nm and 540 nm using a UV-visible spectrophotometer (UV1100, Shanghai Meipuda Instrument Co., Shanghai, China) to analyze the protein and reducing sugar content, respectively. These measurements indicated that the concentration of these substances within the mycelium reflected the stability of the cell membrane in the strain. To quantify protein content, a standard curve was generated by plotting the protein mass (*x*-axis) against the absorbance (*y*-axis), resulting in the regression equation y = 9.66667 × 10^−4^ + 0.00249x, R^2^ = 0.99973. Similarly, the glucose concentration was plotted against the absorbance to generate a standard curve for reducing sugar content, yielding the regression equation y = 0.51811x − 0.02521, R^2^ = 0.99757.

#### 2.7.4. Malondialdehyde (MDA) Content

The supernatant was prepared as described in Section 2.7.1. A 100 μL portion of the supernatant was mixed with 200 μL of the MDA working solution, then heated at 100 °C for 15 min, and subsequently cooled to 25 °C with cold water. The mixture was subjected to centrifugation at 1000× *g* for 10 min, and the absorbance of the supernatant was measured at 450, 532, and 600 nm using a UV–Visible spectrophotometer (UV1100; Shanghai Meipuda Instrument Co., Shanghai, China).

#### 2.7.5. SOD Content

A commercial assay kit (Nanjing Jiancheng Bioengineering Institute, Nanjing, China) was employed to measure the production of SOD. *A. flavus* spores were mixed into liquefied PDA and cultured at 170× *g* for 2 days at 28 °C. Subsequently, CEO was added to the medium at concentrations of 1.56, 0.78, 0.39, and 0.20 g/L. The culture flasks were sealed and maintained in a vibrating incubator at 28 °C for 3 days. Following incubation, the mycelia were harvested, separated on agar, and washed three times. The mycelia were blended in a cold-water bath and centrifuged to collect the resulting supernatant. The absorbance of the supernatant was passively assessed at 550 nm using a UV-Visiblespectrophotometer (UV1100; Shanghai Meipuda Instrument Co., Shanghai, China).

### 2.8. Statistical Analyses

All results are presented as the mean ± standard error of the mean (SEM) from three independent experiments. Statistical analysis was performed using SPSS 27.0 for Windows. A one-way analysis of variance (ANOVA) was conducted to assess differences among groups. If significant differences were found, post-hoc multiple comparisons were performed using Tukey’s Honest Significant Difference (HSD) test to determine which groups differed. Statistical significance was set at *p* < 0.05.

## 3. Results

### 3.1. Microscopic Analysis of Aspergillus Species in Contaminated Walnut Samples

**Figure 1:**
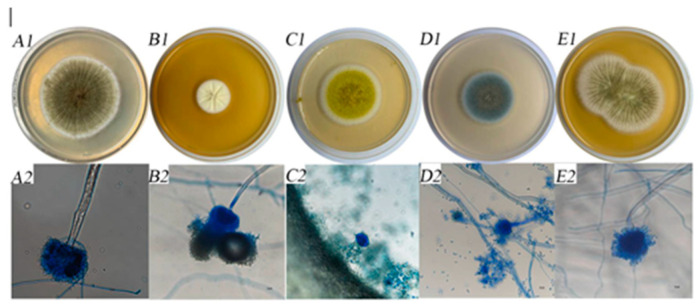
Colony morphology and microscopic morphological characteristics of strains. (**A1**) Colony morphology of strain C10. (**A2**) Microscopic morphological characteristics of strain C10. (**B1**) Colony morphology of strain C11. (**B2**) Microscopic morphological characteristics of strain C11. (**C1**) Colony morphology of strain C14. (**C2**) Microscopic morphological characteristics of strain C14. (**D1**) Colony morphology of strain C16. (**D2**) Microscopic morphological characteristics of strain C16. (**E1**) Colony morphology of strain C19. (**E2**) Microscopic morphological characteristics of strain C19.

A comparison of morphology was performed using CA medium and microscopy following the *Fungal Identification Manual* [26,27]. Figure 1 shows that the cultures were classified into a minimum of five distinct fungal genera. The colony size, surface characteristics, spore formation, and fungal conidia matched the descriptions provided by previous authors [32].

### 3.2. Identification of Fungal Isolates via ITS Sequence Analysis

**Figure 2:**
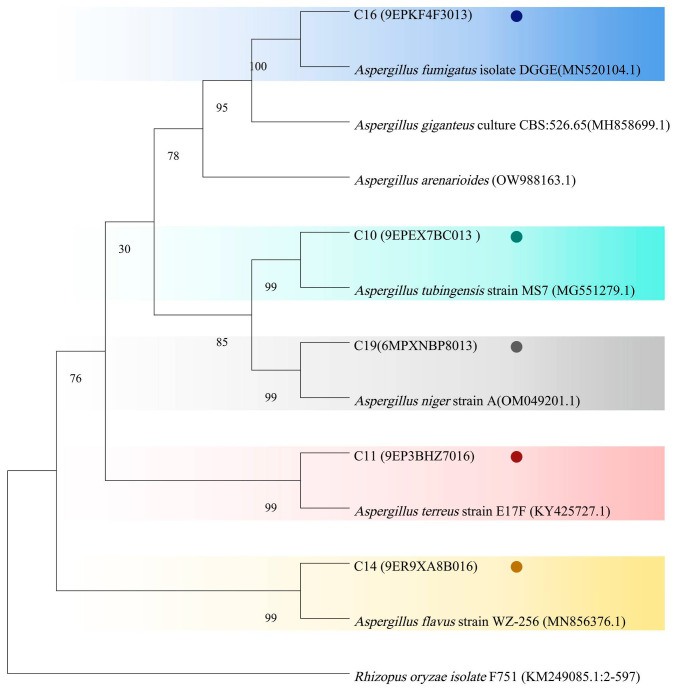
A phylogenetic tree of moldy walnut fungus based on ITS sequences. The numbers in parentheses represent the GenBank accession numbers for the 5.8S rRNA gene sequences of those strains. Bootstrap values at the branching points are expressed as percentages of 1000 replications.

A total of 358 isolates were examined. The amplification of the ITS region with the primers ITS1 and ITS4 resulted in fragments that were roughly 500 to 750 base pairs in length for all five isolates, as verified through gel electrophoresis. The resulting fragments were sequenced and then analyzed against reference data from the GenBank database utilizing the BLAST method. Additionally, ITS sequences from 358 different species were uploaded to GenBank to aid in the identification of these isolates. Every isolate exhibited over 99% similarity of nucleotides to established fungal sequences, enabling accurate species identification. While some isolates showed over 99% similarity of nucleotides with multiple *Aspergillus* spp., the combination of ITS sequence data and morphological characteristics allowed for more accurate species identification. From the 358 isolates, five representative strains were selected to construct a phylogenetic tree. Figure 2 presents a tree diagram representing evolutionary relationships generated using the neighbor-joining method, based on ITS sequences retrieved from GenBank for various fungal species. Five strains of *Aspergillus* spp. were isolated from decaying walnuts. In all five species, the terminal branches exhibited strong support, with high self-expansion values. For example, isolate C14 showed 99% identity with *A. flavus* in the ITS sequence analysis. Morphologically, C14 exhibited typical characteristics of *A. flavus*, such as conidial heads with a ball shape and a yellow–green color on PDA, which supported the identification. According to the analysis of evolutionary relationships, the five strains were recognized as *Aspergillus tubingensis* (C10), *Aspergillus terreus* (C11), *A. flavus* (C14), *Aspergillus fumgiatus* (C16), and *Aspergillus niger* (C19).

### 3.3. Pathogenicity Testing

**Figure 3:**
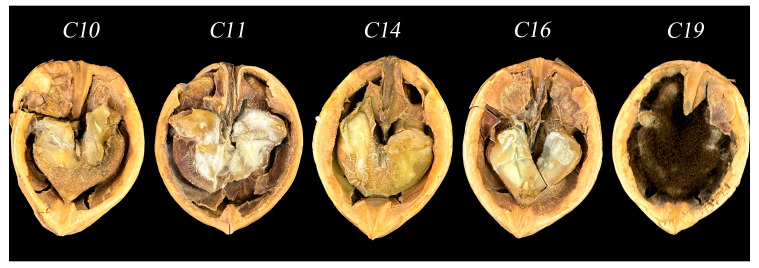
Walnuts were grafted back onto *Aspergillus* spp. strains, such as C10 (*A. tubingensis*), C11 (*A. terreus*), C14 (*A. flavus*), C16 (*A. fumigatus*), and C19 (*A. niger*).

Freshly prepared and sterilized dried walnuts were inoculated with five purified strains of *Aspergillus* spp. The walnuts inoculated with these strains exhibited similar molds as those stored in the ambient environment, while no molds developed in the uninoculated walnuts. As shown in Figure 3, white mold spots were observed on the surface of walnuts inoculated with strain C10 *(A. tubingensis*). Inoculation with strain C11 (*A. terreus*) resulted in the development of white filamentous mycelium on the surface of walnuts. Yellowish–green mold, accompanied by a strong odor of decay, developed on the surface of walnuts inoculated with strain C14 (*A. flavus*). Green mold grew on the surface of walnuts inoculated with strain C16 (*A. fumigatus*), while walnuts inoculated with strain C19 (*A. niger*) exhibited black mycelium and abundant spore production. Walnuts inoculated with *Mycosphaerella* sp. exhibited mold growth on the surface and inside the fruit, eventually resulting in fruit rot. The molds were re-isolated from the decayed walnuts and cultured on a PDA medium to obtain strains with the same morphological characteristics as the inoculated strains. Furthermore, all five strains of *Aspergillus* were found to cause walnut fruit rot.

### 3.4. Frequency of Aspergillus spp. in Moldy Walnut Samples

Overall, 358 isolates were obtained and categorized into five genera (Table 2). Table 2 presents the percentage distribution and total count of *Aspergillus* species identified from moldy walnut kernels. Every one of the 100 samples examined in this study (100%) showed signs of contamination with *A. flavus*. *A. fumigatus* was present in 93 samples (93%), and *A. niger* was isolated from 78 samples (78%). In contrast, *A. terreus* and *A. tubingensis* were detected in only 47% and 40% of the samples, respectively. The number of isolates of *A. flavus* (100), *A. fumigatus* (93), and *A. niger* (78) accounted for 27.93%, 25.97%, and 21.78% of the overall count of isolates, respectively. In contrast, *A. terreus* (47) and *A. tubingensis* (40) demonstrated smaller proportions at 13.12% and 11.17% of the total isolates, respectively.

*Aspergillus* and *Penicillium* are the main storage fungi that commonly infect walnuts stored in unfavorable conditions, including elevated temperatures and moisture levels [33]. Based on these findings, we proposed that *A. flavus* is the dominant pathogenic fungus in walnuts stored in Shanxi, China.

### 3.5. Antifungal Activities of CEO

*A. flavus* (strain C14) exhibited a high frequency and quantity in contaminated walnut samples and was one of the primary fungi identified in the study. Therefore, *A. flavus* was selected as the experimental strain.

#### 3.5.1. CEO Inhibits Mycelial Growth

**Figure 4:**
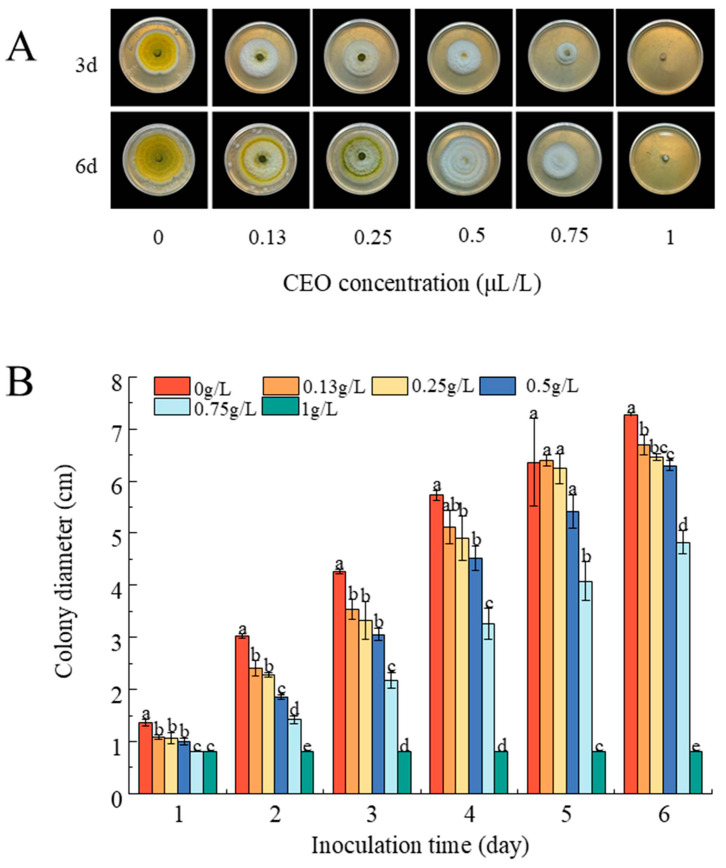
The inhibitory effect of CEO on the mycelial growth of fungi. (**A**) The in vitro antifungal efficacy of CEO at various concentrations (0, 0.13, 0.25, 0.5, 0.75, and 1 g/L) against *A. flavus*. (**B**) The effects of different concentrations of CEO on the colony diameter of *A. flavus*. Different letters (a, b, c, d, and e) above the bars indicate significant differences between groups as determined by one-way ANOVA followed by Tukey’s HSD test (*p* < 0.05).

Figure 4A shows that CEO powerfully curbed the growth of *A. flavus* in a dose-dependent manner. The size of the colony reduced progressively with the increase in CEO concentration. After 3 days, the colony diameters were 3.54, 3.33, 3.05, 2.17, 0.8, and 0.8 cm at CEO concentrations of 0.13, 0.25, 0.5, 0.75, and 1 g/L, respectively, while the control had a diameter of 4.26 cm. The decrease in colony diameter was significant, with increasing CEO concentrations (Figure 4B). After 6 days of incubation, the colony diameter of *A. flavus* treated with 1 g/L CEO did not increase. These data show the concentration-dependent bacteriostatic effects of CEO.

#### 3.5.2. MIC and MFC

**Figure 5:**
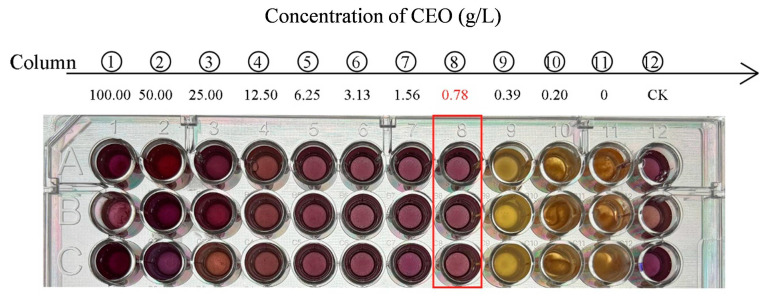
The measurement of the minimum inhibitory concentration of CEO against *A. flavus* using the resazurin reduction method. (①–⑩) Indicate the different CEO concentrations used in the assay, with ① representing the highest concentration and ⑩ representing the lowest. The red box highlights the well where the minimum inhibitory concentration (MIC) was observed, indicating the lowest concentration where growth inhibition was evident.

The MIC and MFC of CEO against *A. flavus* were determined. Figure 5 shows that following 72 h of incubation with constant agitation, the samples treated with CEO concentrations of 0.20 and 0.39 g/L exhibited a color change to a fluorescent, colorless state, showing the existence of living cells [34]. The fungi were then cultivated on PDA plates containing CEO concentrations of 0.78, 1.56, 3.13, 6.25, 12.50, 25, 50, and 100 g/L at 28 °C for 48 h. The growth of fungi was observed on PDA plates with 0.78 g/L CEO, but no growth was observed at 1.56 g/L CEO. Therefore, the MIC and MFC of CEO against *A. flavus* were 0.78 and 1.56 g/L, respectively. These findings indicate that CEO restricts the growth of all *A. flavus* isolates.

### 3.6. Effect of CEO on A. flavus Cell Membrane Permeability

**Figure 6:**
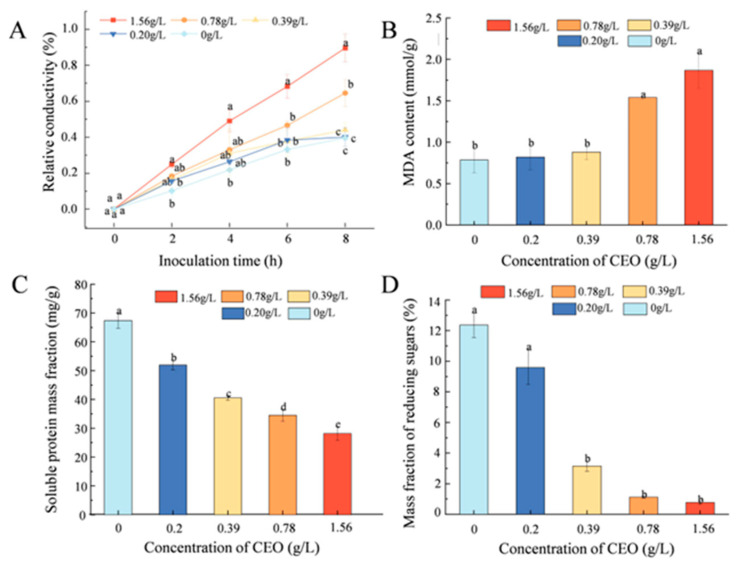
Relative conductivity (**A**), MDA content (**B**), protein content (**C**), and soluble sugar content (**D**) of microbial suspensions before and after CEO treatment. Different letters (a, b, c, d, e) indicate significant differences between groups as determined by one-way ANOVA followed by Tukey’s HSD test (*p* < 0.05).

The permeability of the cell membrane is a key indicator of bacterial inhibition effectiveness [35]. Figure 6A shows that the relative conductivity increased as CEO concentrations increased. After 8 h of treatment, the relative conductivity values for the groups treated with 0.2 g/L and 0.39 g/L of CEO were 90% and 65%, respectively, significantly higher than those in the control group (0 g/L) and in the groups treated with higher concentrations (0.78 and 1.56 g/L) (*p* < 0.05). In summary, the cinnamon essential oil treatment’s propensity to exacerbate the increase in relative conductivity of *A. flavus* cell membranes implies a significant perturbation of the cell membrane structure.

### 3.7. Impact of CEO on the Integrity of Microbial Cell Membranes

The release of cellular contents serves as a significant sign of damage to the cell membrane. Figure 6C shows that the content of soluble protein and sugar in the mycelium decreased as the concentration of CEO increased. The soluble protein levels in the groups treated with CEO at concentrations of 0.39, 0.78, and 1.56 g/L were decreased by 39%, 48%, and 58.19%, respectively, compared to those in the mycelium treated with 0 g/L of CEO, indicating a notable decrease. Additionally, the amount of soluble sugar in the mycelium in the groups treated with 0.39, 0.78, and 1.56 g/L of CEO was considerably less than that of the control group (0 g/L) and the other groups that received CEO (0.2 g/L and 0.39 g/L). The soluble sugar mass content in the mycelium treated with CEO concentrations of 0.39, 0.78, and 1.56 g/L decreased by 9.21%, 11.23%, and 11.78%, respectively, compared to that of the mycelium treated with 0 g/L of CEO. These findings indicate that CEO can rapidly disrupt the plasma membrane of *A. flavus*, resulting in compromised membrane integrity and the subsequent leakage of cellular contents.

### 3.8. Oxidative Stress Evaluation

MDA, a primary byproduct of lipid peroxidation in cell membranes, serves as an indirect marker of oxidative damage [36]. Figure 6B shows that the MDA content increased with rising CEO concentration. The 0.78 g/L and 1.56 g/L treatments resulted in MDA levels of 1.86 and 1.53 mmol/g, respectively, which were considerably greater than those in the control group (*p* < 0.05). In summary, CEO treatment enhanced lipid peroxidation in the cell membranes of *A. flavus*.

**Figure 7:**
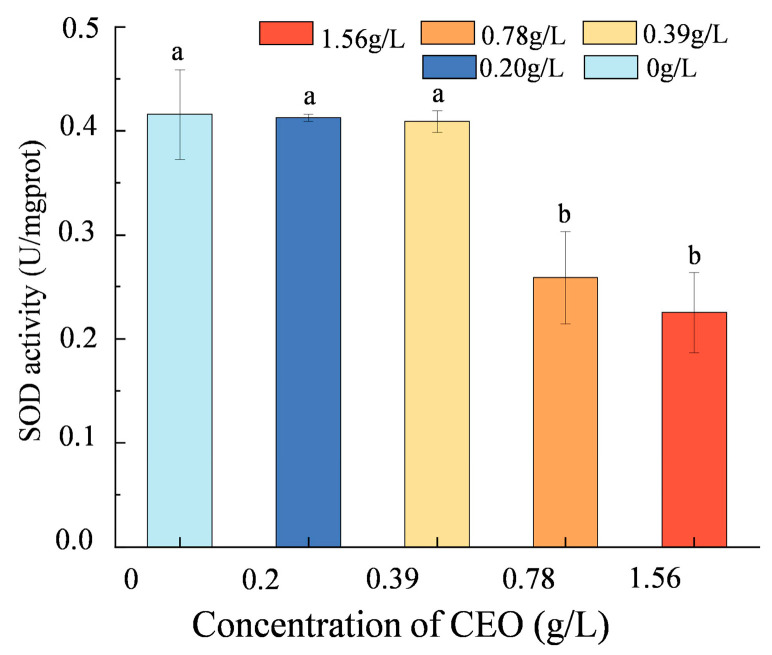
The effect of different concentrations of cinnamon essential oil on the SOD activity of *A. flavus.* Different letters (a,b) indicate significant differences between groups as determined by one-way ANOVA followed by Tukey’s HSD test (*p* < 0.05).

SOD serves as an indicator of the degree of damage caused by lipid peroxidation in cell membranes. To investigate if CEO damages the cell membrane of pathogenic fungi, we assessed the changes in SOD activity of mycelium after treatment with CEO (Figure 7). At CEO concentrations above 0.78 g/L, the SOD activity in *A. flavus* was considerably lower than that of the control group (*p* < 0.05), signaling membrane injury. This suggests that CEO inhibits the activity of SOD in *A. flavus*. The observed changes in SOD activity suggest a potential mechanism by which CEO affects pathogenic fungi. In conclusion, the decrease in SOD activity indicates lipid peroxidation in the fungal cell membrane, leading to membrane damage of the fungus.

## 4. Discussion

In this study, *Aspergillus* species were isolated from moldy walnuts collected in Shanxi, China, and identified using morphological identification and ITS sequence analysis. ITS analysis is widely regarded as a reliable method for fungal identification, especially at the species level, owing to its high accuracy [10]. However, the accuracy of fungal identification may be affected by data quality issues in publicly available ITS sequence databases, including GenBank [37]. Therefore, a combination of morphological identification and molecular biological techniques was employed to enhance identification accuracy.

The results demonstrated that *A. flavus* and *A. fumigatus* were the most prevalent *Aspergillus* species, detected in 100% and over 93% of the samples, respectively. These observations are consistent with earlier studies [38] reporting that *A. flavus* and *A. niger* are the dominant fungi in walnuts. The authors of [39] observed a similar mycobiota linked to nuts and fruits in Saudi Arabia and Iraq. Moreover, this discovery aligns with previous research findings demonstrating that *A. flavus* is prevalent in various nuts and grains, including almonds, peanuts, coffee, and corn [8,11,40]. *A. flavus* is not naturally associated with nut trees and typically infects fruits after harvest [13]. This fungus usually infects crops during the postharvest stage. A slight increase in moisture during storage or transportation can promote mold growth [41]. However, *A. fumigatus* has not been previously identified as a dominant *Aspergillus* spp. in rotting walnuts. Geographic location and environmental differences may be crucial factors in this outcome. *A. fumigatus* exhibits broad ecological adaptations, including a strong capacity to thrive in high temperature and humidity conditions [42]. Furthermore, storage conditions significantly influence fungal growth.

Effective harvesting and postharvest management are essential for achieving the maximum yield of high-quality nuts. However, fungal contamination during storage can significantly affect the quality and safety of the nuts. Recently, consumers have demonstrated increasing resistance to conventional chemical fungicides, suggesting the need for safer, environmentally friendly, and cost-effective alternatives. In response to this demand, we investigated the antifungal efficacy of CEO against *A. flavus*, a highly pathogenic strain isolated locally.

CEO effectively inhibited *A. flavus*, a fungus commonly found in waxberries harvested in Taigu, China. Several studies have demonstrated that essential oils, particularly CEO, exhibit significant antimicrobial activity and can broadly inhibit fungi and bacteria [43,44,45]. The main component of CEO is cinnamaldehyde, a volatile substance with significant antibacterial activity [46]. As a major constituent of CEO, cinnamaldehyde interacts with the cell membrane, where its aldehyde group readily binds to hydrophilic groups on the membrane surface. This interaction disrupts the membrane’s structural integrity and function. Once internalized, cinnamaldehyde interferes with normal cellular metabolism, leading to a loss of cellular homeostasis [47]. Consequently, this disruption results in the leakage of soluble proteins and sugars, as demonstrated in the study, which serves as a clear indication of the compromised membrane integrity and enhanced permeability induced by CEO and its active component, cinnamaldehyde. In addition to CEO, essential oils from *Schinus molle* leaves and Cinnamomum cassia bark have also demonstrated promising antifungal and antileishmanial activities. Major constituents such as spathulenol, β-caryophyllene, caryophyllene oxide, (E)-cinnamaldehyde, cinnamyl acetate, and cis-2-methoxycinnamic acid contribute to these bioactivities [48]. In this study, the observed increase in relative conductivity and the reduction in proteins and reducing sugars in *A. flavus* treated with CEO suggest changes in cell permeability and the disruption of cell membrane integrity. Ref. [31] observed a significant increase in protein levels in microbial suspensions treated with CEO, which differs from the results of this research. This variation could be due to differences in the experimental techniques used. Moreover, in this study, the supernatant was obtained from mycelia rinsed after CEO treatment, while [31] used microbial suspensions. Previous studies have demonstrated that CEO exerts antimicrobial effects by increasing the ability to allow substances to pass through Staphylococcus aureus cell membranes, resulting in the release of intracellular components and compromising membrane integrity [49]. Previous studies have shown that cinnamon essential oil disrupts the cell membrane of *Rhizopus stolonifer*, leading to an increase in extracellular conductivity, which is consistent with the findings of this study [50]. These results align with the outcomes of this research.

CEO also significantly inhibited SOD activity in *A. flavus*, preventing it from scavenging excess superoxide radicals generated within the cell. The accumulation of superoxide radicals led to cell membrane peroxidation. The MDA produced during peroxidation inactivates enzymes and proteins in the membrane, leading to the formation of membrane gaps and thus increasing permeability and impairing membrane functions. These alterations hinder the development and reproduction of microorganisms [30]. The findings of this study align with previous findings, where the decrease in SOD activity indirectly led to increased MDA content. The increase in MDA levels indicates that CEO promotes the oxidation of membrane lipids; this is supported by previous study findings [51]. This study aligns with the findings of Xue et al. [24], who observed that CEO treatment significantly increased MDA levels in *A. flavus*. MDA is generated by the peroxidation of lipid membranes, leading to the formation of cross-links with enzymes, nucleotides, amino acids, and various other molecules. This process leads to the creation of insoluble elements, resulting in structural harm to plasma membranes [52].

This study mainly investigated the inhibitory effect of CEO through in vitro experiments and failed to address the actual storage and transportation conditions. In practice, the storage environment and treatment of pecans and the actual application conditions of CEO may be different, affecting the effectiveness of CEO. Although we indicated that CEO inhibits the growth of *A. flavus* by damaging cell membranes and promoting membrane lipid peroxidation, the specific molecular mechanisms have not been explored in depth. Hence, further research and validation are still needed to elucidate the mechanism by which CEO acts on fungi at the molecular level.

In addition, the potential toxicity of CEO in human food should not be overlooked. Studies have shown that high concentrations of CEO can cause skin irritation and allergic reactions [53]. The coumarin present in CEO may exhibit hepatotoxicity and nephrotoxicity at high concentrations, with prolonged or excessive intake potentially leading to liver and kidney damage [54]. The European Food Safety Authority (EFSA) has established safety limits for coumarin use in food, emphasizing the importance of dilution and appropriate application to minimize potential adverse effects [55]. Despite these concerns, the use of CEO in food is generally considered safe when applied at appropriate concentrations. Strict adherence to these guidelines is essential to ensure the safe consumption of food products containing CEO.

Taken together, the antifungal properties of CEO are due to its potential to harm the plasma membrane and induce peroxidation of membrane lipids. Moreover, CEO constitutes a safe, sustainable substitute for commercial fungicides to control the decay of walnuts after harvest.

## 5. Conclusions

The study investigated *Aspergillus* contamination in moldy walnuts randomly collected from Shanxi Province, identifying the prevailing species as *A. flavus* (100%), *A. fumigatus* (93%), *A. niger* (78%), *A. terreus* (47%), and *A. tubingensis* (40%). The study also investigated the bacteriostatic activity and inhibition mechanisms of CEO against *A. flavus* in walnuts. The findings revealed that the MIC of CEO for *A. flavus* strains was 0.78 g/L, highlighting its potential as an effective agent for microbial control in walnuts and other food products. Additionally, CEO disrupted the structure of microbial cell walls and membranes while significantly increasing intracellular lipid peroxidation. As a result, CEO could be utilized as a natural bactericide in food preservation. The effectiveness of CEO’s anti-microbial properties has only been confirmed in walnuts, and thus its use in a wider range of food matrices for large-scale practical applications requires additional investigation.

## Figures and Tables

**Table 1:** Mean high temperature, mean low temperature (in °C), and total monthly precipitation (in cm) in the Taigu District, Jinzhong City, Shanxi Province, China, June–October 2023.

Months	June	July	August	September	October
Average high temperature (°C)	31	32	31	26	21
Average low temperature (°C)	14	19	18	13	4
Total monthly precipitation (cm)	14.1	16.5	76.3	4.8	17.8

**Table 2:** Frequency and quantity of *Aspergillus* spp. isolates retrieved from moldy walnut samples.

Sample	*A. tubingensis*	*A. terreus*	*A. flavus*	*A. fumigatus*	*A. niger*
detection limit	40	47	100	93	78
Frequency	40%	47%	100%	93%	78%

## Data Availability

The original contributions presented in this study are included in the article. Further inquiries can be directed to the corresponding author.
